# Complete Genome Sequences of Canadian Epidemic Methicillin-Resistant Staphylococcus aureus Strains CMRSA3 and CMRSA6

**DOI:** 10.1128/MRA.00892-18

**Published:** 2018-08-09

**Authors:** Jo-Ann McClure, Steven M. Shideler, Kunyan Zhang

**Affiliations:** a Centre for Antimicrobial Resistance, Alberta Health Services/Calgary Laboratory Services/University of Calgary, Calgary, Alberta, Canada; b Department of Pathology and Laboratory Medicine, University of Calgary, Calgary, Alberta, Canada; c Department of Microbiology, Immunology and Infectious Diseases, University of Calgary, Calgary, Alberta, Canada; d Department of Medicine, University of Calgary, Calgary, Alberta, Canada; e The Calvin, Phoebe and Joan Snyder Institute for Chronic Diseases, University of Calgary, Calgary, Alberta, Canada; Georgia Institute of Technology

## Abstract

Methicillin-resistant Staphylococcus aureus (MRSA) clonal complex 8 (CC8) sequence type 239 (ST239) represents a predominant hospital-associated MRSA sublineage present worldwide. The Canadian epidemic MRSA strains CMRSA3 and CMRSA6 are moderately virulent members of this group but are closely related to the highly virulent strain TW20. Whole-genome sequencing of CMRSA3 and CMRSA6 was conducted to identify genetic determinants associated with their virulence.

## Abstract (precis)

Methicillin-resistant Staphylococcus aureus (MRSA) clonal complex 8 (CC8) sequence type 239 (ST239) represents a predominant hospital-associated MRSA sublineage present worldwide. The Canadian epidemic MRSA strains CMRSA3 and CMRSA6 are moderately virulent members of this group but are closely related to the highly virulent strain TW20.

## ANNOUNCEMENT

The methicillin-resistant Staphylococcus aureus (MRSA) clonal complex 8 (CC8) sequence type 239 (ST239) subgroup represents a predominant health care-associated lineage present worldwide (1, 2). Holden and colleagues (3) reported a highly transmissible and invasive outbreak MRSA strain, TW20 (ST239-t037-MRSA-III-*agr*-I), belonging to this CC8-ST239 sublineage. In Canada, the Canadian epidemic MRSA strains CMRSA3 (ST241-t037-MRSA-III-*agr*-I) and CMRSA6 (ST239-t037-MRSA-III-*agr*-I) are hospital-associated MRSA strains, but they are infrequently isolated and are associated with less severe and/or invasive infections (4). Both strains are closely related to the highly virulent outbreak strain TW20. To identify genetic determinants responsible for the contrasting virulence noted among these strains, the genomes of CMRSA3 and CMRSA6 were sequenced.

The genomes of CMRSA3 and CMRSA6 were sequenced with Pacific Biosciences (PacBio) RS II sequencing technology, using one single-molecule real-time (SMRT) cell, as well as with Illumina MiSeq technology (2 × 250-bp paired ends) using a single nanocartridge. Hybrid assembly was conducted using LoRDEC version 0.9 (5), Canu version 1.7 (6), SPAdes version 3.10.1 (7), Minimap version 2.10 (8), and Pilon version 1.22 (9); the long reads were corrected with short reads, and the corrected long reads were assembled. Gene annotation was done using the NCBI Prokaryotic Genome Annotation Pipeline. PacBio sequencing of CMRSA3 generated 66,867 reads covering 765,486,759 sequenced bases, with an average read length of 11,447 bp (longest read length of 51,749 bp). The estimated genome coverage was 234× with a GC content of 32.97%. Following Illumina sequencing and hybrid assembly, the resulting closed chromosome was 2,958,212 bp and contained 3,122 genes, of which 2,947 were coding sequences (CDSs), 83 were RNA genes, and 92 were pseudogenes. A closed and complete plasmid was also identified in CMRSA3 (pCMRSA3) with a length of 27,354 bp. PacBio sequencing of CMRSA6 generated 57,790 reads covering 626,480,961 sequenced bases, with an average read length of 10,840 bp (longest read length of 53,372 bp). The estimated genome coverage was 193× with a GC content of 32.78%. Following hybrid assembly, the resulting closed chromosome was 3,044,721 bp and contained 3,216 genes, of which 3,037 were CDSs, 79 were RNA genes, and 100 were pseudogenes.

Several genetic components associated with virulence were detected on the chromosomes of both CMRSA3 and CMRSA6. The genome of CMRSA3 carries two prophages, including ΦSa6 (with no known virulence-related genes) and ΦSa3 (bearing the staphylococcal complement inhibitor [SCIN], enterotoxin A, and chemotaxis inhibitory protein), as well as the pathogenicity island SaPI1 (with enterotoxins K and Q). On CMRSA3’s plasmid, the *qacA* gene (conferring antiseptic resistance) and the mercury and cadmium operons (conferring resistance to the heavy metals mercury and cadmium, respectively) were identified. Similar to CMRSA3, the genome of CMRSA6 also carries ΦSa6 (with no known virulence-related genes), ΦSa3 (with staphylokinase, enterotoxin A, and SCIN), and SaPI1 (with enterotoxins K and Q). CMRSA6 was also found to carry a ΦSPβ-like prophage that contains genes related to resistance and bacterial persistence. A more detailed analysis is under way comparing the genomes of these variant virulence strains, with the goal of identifying determinants that contribute to the pathogenesis of the MRSA ST239 sublineage.

### Data availability.

The chromosomal genome sequences have been deposited at GenBank under the accession numbers CP029685 (CMRSA3), MH470063 (pCMRSA3), and CP027788 (CMRSA6).
